# Rapid Detection of Panton–Valentine Leukocidin Production in Clinical Isolates of *Staphylococcus aureus* from Saxony and Brandenburg and Their Molecular Characterisation †

**DOI:** 10.3390/pathogens14030238

**Published:** 2025-03-01

**Authors:** Elke Müller, Stefan Monecke, Marc Armengol Porta, Marco Vinicio Narvaez Encalada, Annett Reissig, Lukas Rüttiger, Percy Schröttner, Ilona Schwede, Hans-Herman Söffing, Alexander Thürmer, Ralf Ehricht

**Affiliations:** 1 Leibniz Institute of Photonic Technology (Leibniz-IPHT), Leibniz Center for Photonics in Infection Research (LPI), Germany and InfectoGnostics Research Campus, 07745 Jena, Germany; 2 InfectoGnostics Research Campus, Centre for Applied Research, 07745 Jena, Germany; 3 Medizinisches Labor Ostsachsen MVZ GbR, 01067 Dresden, Germany; 4 Städtisches Krankenhaus Dresden, 01067 Dresden, Germany; 5 Senova Gesellschaft für Biowissenschaft und Technik mbH, 99427 Weimar, Germany; 6 Institute for Medical Microbiology and Virology, Faculty of Medicine and University Hospital “Carl Gustav Carus”, Technische Universität Dresden, 01307 Dresden, Germany; 7 Institute for Clinical Chemistry and Laboratory Medicine, Faculty of Medicine and University Hospital “Carl Gustav Carus”, Technische Universität Dresden, 01307 Dresden, Germany; 8 IMD Labor Oderland GmbH, 15230 Frankfurt (Oder), Germany; 9 MVZ Medizinische Labore Dessau Kassel GmbH, 06847 Dessau, Germany; 10 Institute of Physical Chemistry, Friedrich Schiller University Jena, 07745 Jena, Germany

**Keywords:** *Staphylococcus aureus*, community-acquired MRSA, Panton–Valentine leukocidin (PVL), lateral flow test

## Abstract

Panton–Valentine leukocidin (PVL) is a staphylococcal toxin associated with chronic/recurrent skin and soft tissue infections (SSTIs) and necrotizing pneumonia. Its detection in clinical isolates of *Staphylococcus aureus* warrants aggressive therapy and infection control measures. However, PVL detection relies on molecular methods of limited use, especially in outpatient or resource-poor settings. In order to aid the development of a lateral flow (LF) test for PVL, clinical isolates from SSTIs were collected in 2020/21 at three laboratories in two cities in the Eastern part of Germany. After the exclusion of duplicate and serial isolates, 83 isolates were eligible. These were tested using an experimental LF test for PVL production. They were also characterized using DNA microarrays, facilitating the detection of virulence and resistance markers as well as the assignment to clonal complexes and epidemic/pandemic strains. Thirty-nine isolates (47%) were PVL-positive, and the LF results were in 81 cases (97.6%) concordant with genotyping. One false-positive and one false-negative case were observed. This translated into a diagnostic sensitivity of 0.974 and a diagnostic specificity of 0.977. The most common PVL-positive MSSA lineages were CC152 (n = 6), CC121 (n = 4), and CC5 and CC30 (each n = 2). Thirty isolates (36%) were *mecA*-positive. The MRSA rate among PVL-negatives was 20% (nine isolates), but among the PVL-positives, it was as high as 54% (n = 21). The most common PVL-MRSA strains were CC398-MRSA-VT (n = 5), CC5-MRSA-IV “Sri Lanka Clone” (n = 4), CC8-MRSA-[*mec* IV+Hg] “Latin American USA300” (n = 4), and CC22-MRSA-IV (PVL+/*tst*+) (n = 2). While the PVL rate was similar just like the German isolates from a previous study a decade before, the MRSA rate among PVL-positives was clearly higher. All PVL-MRSA strains detected, as well as the most common methicillin-susceptible lineage (CC152), are known to be common locally in other parts of the world, and might, thus, be regarded as travel-associated. Therefore, patients with suspected PVL-associated disease should be asked for their history of travel or migration, and, in case of hospitalization, they should be treated as MRSA cases until proven otherwise.

## 1. Introduction

*Staphylococcus (S.) aureus* is a ubiquitous Gram-positive bacterium that colonizes or infects humans as well as a wide variety of mammal and bird species. It can cause various skin and soft tissue infections (SSTIs), osteomyelitis, pneumonia, sepsis, and toxin-mediated conditions, such as toxic shock syndrome and food intoxications. Many strains of this bacterium carry resistance genes on mobile genetic elements, with the most notable example being *mecA* (and/or the related *mecC*, which, however, has never been found in PVL-positive strains), localized on SCC*mec* elements [1,2,3,4]. This gene encodes an alternative β-lactam-binding protein and, thus, causes resistance to most β-lactam antibiotics. Strains that carry it are named methicillin-resistant *S. aureus*, or MRSA, as methicillin is an indicator substance for this phenomenon. *S. aureus* strains also can variably harbour mobile genetic elements that include genes for various toxins and virulence factors.

One virulence factor that often plays a role in the development of SSTIs and pneumonia in humans is the Panton–Valentine leukocidin (PVL, [5,6,7,8]). It is encoded by two neighbouring genes located together on prophages [9,10,11] integrated invariably at the same position into the bacterial chromosome (*lukS-PV* and *lukF-PV*, GenBank BA000033.2:MW1378 and MW1379; [12]). This genetic arrangement allows for the horizontal transfer of the PVL toxin between different *S. aureus* strains through a process called lysogenic conversion. When the prophage is induced, it can replicate lyse, the host cell, and infect other *S. aureus* bacteria, potentially transferring the PVL genes to new staphylococcal cells. This mechanism of transmission contributes to the spread of PVL across diverse and rather unrelated lineages of *S. aureus*, and even into related staphylococcal species [13]. Together, the molecules of the two components of leukocidin genes form polymeric pores in leukocyte membranes. This kills these cells in a highly specific manner resulting in the release of cytokines [8,14] which, in turn, attracts more leukocytes. While PVL is rather specific for human leukocytes [15], they are structurally and functionally similar to leukocidins, and can be detected in *S. aureus* strains from various animal species. *LukM/lukF-P83* [8] is known for ruminant strains causing mastitis and for a strain associated with dermatitis in tree squirrels [16,17]. Another set of leukocidin genes, called *lukP/lukQ*, has been identified in *S. aureus* strains isolated from horses [18,19] while small ruminants harbour strains with a chimeric leukocidin derived from *lukP/lukQ* and *lukM/lukF-P83* [19]. Yet, another leukocidin (*lukF/S-BV*) which is closely related to PVL was recently discovered in *S. aureus* from Eurasian beavers [20]. As all are carried on prophages, this suggests that phage infections of *S. aureus* may predetermine both host specificity and clinical manifestations of *S. aureus* infection in its vertebrate host.

PVL-associated diseases in humans occur particularly in immunocompetent young, previously healthy patients. Clinically, they manifest as recurrent or chronic SSTIs, abscesses, furunculosis, as well as so-called “spider-bite lesions”. Case clusters or outbreaks frequently affect families, people who are confined to close proximity to each other, such as inmates of prisons or recruits in army barracks, or those who share possibly contaminated items such as towels, diving masks, breathing devices, etc. PVL-associated disease also includes abscessing or necrotizing pneumonia that can be a complication of viral respiratory tract infections and has a mortality rate of as high as 40% [5,7,21].

While PVL is present only in about a small percentage of *S. aureus* isolates [22], it can be detected much more frequently in isolates originating from SSTIs [5,23]. There used to be large differences in the prevalence of PVL depending on geographical regions, with PVL-positive strains being common in, for instance, Northern America, India, the greater Middle East, sub-Saharan Africa [24], and Australia while being rare in Northern and Central Europe [22], although this might change with the spread of PVL-MRSA. Most interestingly, the prevalence of anti-PVL antibodies exhibits a notable north–south gradient, with significantly higher levels observed in African populations compared to European ones [25]. This serological difference reflects the varying exposure to PVL-positive *S. aureus* strains across geographical regions, and it might also contribute to the apparent rarity of severe/fatal PVL infections in regions where PVL-positive staphylococci are common.

In the 1940s and 1960s, and from the mid-1990s to the present day, there have been worldwide clusters of furunculosis caused by PVL-positive strains. While the first known strains were sensitive to all antibiotics (typical representatives are the laboratory strains ATCC23925 [26] and “Oxford *Staphylococcus*” [27]), the strains that dominated in the 1960s (phage type 80/81) and thereafter were mostly penicillinase-positive. Epidemiological studies from many countries around the world show that PVL-positive MRSAs are currently spreading. There are distinct strains that predominate in different geographic regions. In particular, the strain known as “USA300” became very common in the USA during the last decades, with approximately 60% of all community-acquired skin and soft tissue infections caused by *S. aureus* being attributable to this strain [28,29]. Other strains originate from the Indian subcontinent [30,31,32,33] or Australia [34,35,36,37]. In Europe, a wide variety of different strains of PVL-MRSA occur, and many cases have travel histories or family ties in regions where PVL-MRSA is more common [38,39,40,41,42].

Because of the higher virulence of PVL-positive strains, a more aggressive approach to therapy is warranted. This includes a more liberal administration of antibiotics in addition to incision and drainage of abscesses, and it also includes infection control and eradication measures akin to those applicable in the case of MRSA colonization/infection [43,44]. PVL-MRSA, obviously, are more complicated to treat, and for that reason, it is discussed whether infections with PVL-MRSA should be declared a notifiable disease. In the German state of Saxony, this is already the case [45,46]. However, infection control measures are hindered by the lack of a convenient diagnostic tests. PVL detection is currently essentially limited to molecular methods. These normally are performed only in specialized laboratories with dedicated hardware and trained personnel, and they require complex sample preparation. Most cases, however, are seen in the outpatient setting, where molecular tests are not readily available. A referral of isolates to reference laboratories usually requires too much time until results are available, so that these results do not have a significant impact on the case management anymore. In addition, costs play a major role as general practitioners have limited budgets for laboratory tests while many patients come from marginalized sections of the population, such as prison inmates, substance users or refugees.

For these reasons, efforts are underway to develop a lateral flow assay for the detection of PVL from *S. aureus* cultures. Lateral flow tests have achieved some popularity during the COVID-19 pandemic, so a short description of the principle should be sufficient. The test device contains target-specific antibodies, IgG antibodies coated to particles, and other target-specific IgG antibodies coated on a membrane. During testing, the molecules from the sample react with the antibodies coating the particles. The mixture migrates upward on the membrane chromatographically, by capillary force, to react with captured antibodies on the membrane and generate a coloured line. Its presence in the test region indicates a positive result, while its absence indicates a negative result. To serve as a procedural control, a coloured line will always appear in the control line region.

In order to aid the development of a lateral flow test for PVL, clinical isolates from SSTI were collected in 2020/21 at two laboratories in Eastern parts of Germany. Isolates were tested using an early experimental stage of the LF assay, and they were genotyped in parallel using a DNA-based microarray assay that allowed not only to confirm or to rule out the presence of PVL genes and *mecA*, but also to assign a given isolate to clonal complexes (CC, i.e., phylogenetic lineages within *S. aureus*) and known epidemic strains.

## 2. Materials and Methods

### 2.1. Isolates

Isolates were collected in 2020/21 at two laboratories in Dresden, Saxony, Germany, and at one in Frankfurt/Oder, Brandenburg, Germany. Clinical partners were advised to submit isolates originating from furunculosis or carbuncles, cutaneous abscesses, other conspicuous or severe skin and soft tissue infections, such as mastitis or necrotizing fasciitis, chronically purulent and painful “spider bites”, recurrent or chronic skin- and soft tissue infections (with the exception of diabetic foot ulcers), as well as isolates from cases of necrotizing community-acquired pneumonia, including cases associated with influenza. After the exclusion of duplicate and serial isolates, eighty-three isolates were eligible for study. Twenty-six originated from a communal/city hospital in Dresden (Dresden Friedrichstadt), thirty from the University Hospital of Dresden, and twenty-seven from a diagnostic laboratory serving healthcare providers in and around Frankfurt/Oder.

### 2.2. Antibodies and Lateral Flow Test Productions

Antibodies were generated using the mouse cell hybridoma method, according to Kohler and Milstein [47] using the recombinant *lukF-PV* gene product as an antigen. The manufacturing of the lateral flow tests was performed in Senova, Weimar, Germany.

### 2.3. Lateral Flow Test

*S. aureus* isolates were incubated on a Columbia blood agar overnight for up to 24 h at 37 °C ± 2 °C. If contaminations or mixed cultures were suspected, cultures were re-cloned. Catalase and coagulase/clumping factor tests or species identification by MALDI-TOF were performed to confirm *S. aureus.* One inoculation loop (ca. 1 µL) full of culture material was inoculated into 300 µL of a buffer (phosphate-buffered saline, PBS, plus 0.05% *v*/*v* Tween20) and the mixture was shortly vortexed. Then, it was centrifuged in order to sediment the cells (30 s at 2000× *g*). In total, 100 µL of the supernatant was pipetted onto the sample well of the test device, which was then incubated for 10 min at room temperature. The results were visually read: the presence of both test and control lines was considered positive, while the presence of the control line alone was considered negative. An absence of the control line was interpreted as invalid. The test results for the individual isolates are shown in Supplemental file S1.

### 2.4. Microarray Procedures

The molecular characterization was carried out using the InterArray Genotyping Kit *S. aureus* (InterArray GmbH, Bad Langensalza, Germany) for the detection of species markers as well as of virulence and resistance genes. Probes, primers, the test principle, and procedures were previously described in detail ([48,49,50]; https://doi.org/10.1371/journal.pone.0017936.s001; accessed on 20 February 2025). In short, a multiplexed primer elongation reaction was performed on sample DNA from pure culture amplifying and labelling specific fragments of the target genes. These were hybridized to the microarray carrying specific probes in a predefined pattern. Hybridization was detected using an enzymatic precipitation reaction triggered by the labels incorporated into the amplicons. The microarray harboured probes for both, *lukS-PV* and *lukF-PV*, thus facilitating the molecular confirmation of the lateral flow immunoassay as well as the detection of other virulence genes, resistance genes, and markers that were used for the assignment to CCs and strains. Microarray images were taken, and analysis was carried out using a dedicated reader and software (InterArray GmbH; software versions 1.0.7 gamma to 1.1.8). The full array data are shown in Supplemental file S1.

## 3. Results

One hundred isolates were collected, but after the exclusion of duplicate and serial isolates, eighty-three isolates were finally eligible for the study. Thirty-nine isolates (47%) were PVL-positive. Microarray-based genotyping revealed one false-negative result and one (weakly) false-positive result. This translated into a diagnostic sensitivity of 0.974, a diagnostic specificity of 0.977, a positive prediction value of 0.974, a negative prediction value of 0.977, and an accuracy of 0.976.

The PVL- and *mecA*-status, as well as CC and strain affiliations of the isolates, are summarized in Table 1. Thirty isolates (36.1%) were *mecA*-positive. In total, 35 isolates (42.2% of the total) were PVL-negative MSSA, and 9 isolates (10.8%) were PVL-negative MRSA. The MRSA rate among PVL-negatives was thus 20.5%. The most common lineages among PVL-negative MSSA were CC15, CC22, CC30, and CC45. PVL-negative MRSA included a variety of different strains; see Discussion. Eighteen isolates (21.7% of the total) were PVL-positive MSSA and twenty-one (25.3%) were PVL-positive MRSA. The MRSA rate among PVL-positives was as high as 54% (n = 21).

The most common PVL-positive MSSA lineages were CC152 (n = 6), CC121 (n = 4), and CC5 and CC30 (each n = 2). The most common PVL-MRSA strains were CC398-MRSA-VT (n = 5), CC5-MRSA-IV “Sri Lanka Clone” (n = 4), CC8-MRSA-[IV+Hg] “Latin American USA300” (n = 4), and CC22-MRSA-IV (PVL+/*tst*+) (n = 2). Other PVL-MRSA were CC1-MRSA-[V+*fus+ccrAB1*], CC8-MRSA-[IV+ACME] “USA300”, CC30-MRSA-IV “WSPP Clone”, CC88-MRSA-IV, CC152-MRSA-IV, and –[V+*fus*], with one isolate each (see Discussion for details).

The alternate gene for methicillin resistance, *mecC*, was not found. The SCC*mec*-borne gene for fusidic acid resistance, *fusC*, was detected in nine isolates, all of them being MRSA, two being PVL-positive. The mupirocin resistance gene *mupA* was found twice, in a PVL-MRSA (of the “USA300” strain) and in a PVL-negative MSSA. The prevalence rates for genes responsible for resistance to antibiotic compounds as detected by microarray are listed in Table 2. Given that first-line antibiotics for the treatment of PVL-associated SSTI include oral trimethoprim-sulfamethoxazole, clindamycin, and doxycycline [44], the high rates of carriage of relevant resistance genes, especially among PVL-MRSA (*dfrA*, 19%; *tet*-genes, 47.6%; *erm*-genes, 52.4%), warrant attention.

## 4. Discussion

The experimental performance, as confirmed by the genotypic experiments of the lateral flow assay, was fairly sufficient, given that this was an experimental, pre-production batch. A single false-negative result might be attributed to poor or absent in vitro toxin production. It is known that in vitro expression levels of the toxin vary widely [51,52], and that, in addition to culture conditions [53], mutations in regulatory genes might affect it [52,54]. Thus, it is not surprising that an isolate might be found in which *lukS/F-PVL* genes were present, although the protein was undetectable. The false-positive result remained unexplained. Further studies might focus on culture conditions, i.e., on growth media composition and supplements, as well as on optimization of the assay and its procedures. In general, the lateral flow test provides a suitable tool for a rapid and inexpensive detection of PVL by clinical isolates of *S. aureus*. This might appeal to laboratories that do not routinely perform molecular tests. It also might shorten the time until a test result is available to the care provider, especially in settings where it was otherwise necessary to ship suspected isolates to central reference laboratories.

**Figure 1:**
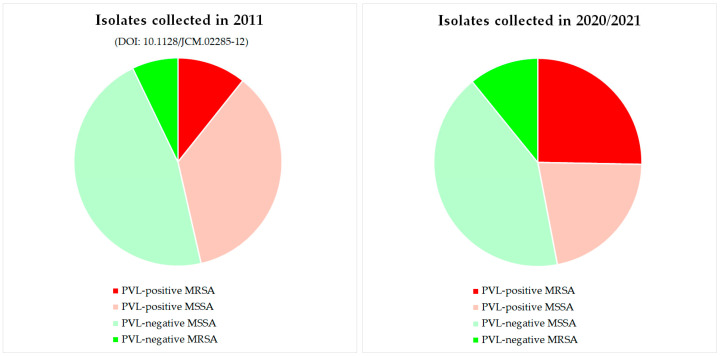
Comparison to the results of the German branch of the previous study [55].

A very similar study was conducted by the authors roughly a decade ago [55], and a direct comparison of the present results to the ones of the German branch of this previous study is interesting (Figure 1).

Then, fifty-six isolates were characterized that were collected in Dresden or submitted by cooperating centres to the Dresden laboratory, using the same inclusion criteria. Twenty-six (46.4%) of these isolates were PVL-negative MSSA, and four (7.1%) were PVL-negative MRSA. Twenty isolates (35.7%) were PVL-positive MSSA, and six (10.7%) proved to be PVL-positive MRSA. Thus, it can be assumed that the “ecological niche” for PVL-positives remained unchanged during that decade. However, a clear shift occurred from PVL-positive MSSA to PVL-positive MRSA, whose share of the total increased from about 10% to about 25%. This is an interesting observation. From the eastern part of Germany, no large outbreak or epidemic situation of community-associated MRSA (ca-MRSA) and/or PVL-MRSA has been described to date. The only major outbreak in Germany of such a clone was observed in the early 2000s in Bavaria [56]. Since then, PVL-positive MRSA has been uncommon, and there is no single predominant clone such as “USA300” in the USA [57,58,59,60]. Contrarily, there was, for decades, a number of different, sporadic strains, which can easily be explained by random importations affecting travellers and possibly immediate contacts, such as family members, in absence of an ongoing epidemic transmission in the local community [57,58,59,60].

The most common methicillin-susceptible/PVL-positive lineages in this study were CC121 and CC152. The former can be found essentially anywhere in the world. The latter is known to be common in Sub-Saharan Africa [61,62,63,64,65,66].

All PVL-MRSA strains detected are known to locally predominate in other parts of the world, and thus, all might be regarded as travel-associated. The most common PVL-MRSA in this study was CC398-MRSA-VT (PVL+). While PVL-negative CC398-MRSA became well-known as livestock-associated MRSA lineage in Western Europe, PVL-positive specimens of CC398 were frequently described from Southeast Asia and from travellers with a corresponding travel history. Touristic travel might, here again, have played a role in the importation of that strain, as well as the fact that the Eastern German Federal States are home to a large community of Vietnamese descent.

Some of the strains observed indicate epidemiological connections to the Middle East and could be regarded as associated either with travel to popular tourist destinations or to migration from Middle Eastern zones of political upheaval. These strains include CC1-MRSA-[V+*fus+ccrAB1* (PVL+), which has been observed by the authors as well as by others in the greater Middle East, especially Egypt [67,68]. CC22-MRSA-IV (PVL+/*tst*+) was apparently first observed in Iran [69], but more recently, it can be found elsewhere in the Gulf region [70], and it is spreading into Asia where it has already been detected in China [71] and Nepal [72]. CC88-MRSA-IV (PVL+) and CC152-MRSA-[V/VT+*fus*] (PVL+) also have been observed in the Gulf region and Egypt [67,68]. C152-MRSA-IV is a sporadic strain with anecdotal evidence pointing towards Eastern Africa (GenBank accession numbers CP141387.1, CP141398.1, CP141523.1) and the Middle East [70].

CC8-MRSA-[IV+Hg] (PVL+) “Latin American USA300” occurs in Southern America and Spain [73,74]. CC8-MRSA-[IV+ACME] (PVL+) “USA300” is common in Northern America and, to a lesser extent, in other Anglophone countries of the world. CC30-MRSA-IV (PVL+) “WSPP Clone” originated from the West Pacific region [75,76] but was meanwhile observed, although sporadically, in many countries. CC5-MRSA-IV (PVL+) “Sri Lanka Clone” is an emerging clone that was first described from Sri Lanka but that recently caused outbreaks across several European countries [77,78]. For all these strains, travel and tourism might have played a role in the importation of these strains into the study settings.

Regarding PVL-negative MRSA strains, one isolate belonged to CC22-MRSA-IV. This strain (“UK EMRSA15” or locally known as “Barnim epidemic strain”, [79]) is a common healthcare-associated MRSA strain in Eastern Germany [58]. One isolate was the “European CC1-MRSA-IV strain”, a strain originating from Southeastern Europe that spread to several Central/Western European countries, possibly aided by its ability to evade a frequently used molecular screening test [80,81,82]. The detection of as many as three isolates of CC5-MRSA-[IV+*fus+ccrAB*], “Maltese Clone”, all from Frankfurt/Oder, is somewhat startling. This strain was first described in Malta [83], which is a tourist destination, but it was also observed in the Kingdom of Saudi Arabia [84,85] and the UAE [86], possibly indicating a wider distribution in the Middle East. Thus, for this strain, as well as for the other *fusC*-positives (because *fusC* in MRSA appears to be common in the greater Middle East [67,68,87,88,89,90,91]), a connection to touristic travel and/or migration might be assumed.

Therefore, patients with suspected PVL-associated disease, and patients with any “ca-MRSA”—regardless of PVL-status—should be asked for their travel histories. Furthermore, patients with suspected PVL-associated disease should, in case of hospitalization, be treated as MRSA cases until proven otherwise, because the odds of indeed carrying MRSA are above 50%.

Given the increase in PVL-MRSA and their clinical relevance, a mandatory notification to public health authorities should be considered. In Saxony, PVL-MRSA infections are already a notifiable disease [45,46], but this is not the case in other German Federal States. The main obstacle to stringent surveillance of PVL-positive *S. aureus* and PVL-MRSA is currently the lack of a quick and cheap assay for PVL. After all, most cases are seen in outpatient settings rather than in tertiary care centres, so that a lack of availability of laboratory infrastructure and molecular diagnostics impedes testing for PVL genes. An easy-to-use non-molecular test such as a lateral flow assay out of a primary culture might thus prove very helpful.

## 5. Conclusions

In conclusion, a lateral flow test for a time- and cost-efficient detection of PVL in overnight cultures of *S. aureus* might prove helpful to clinical laboratories, especially in settings where molecular tests are not feasible and where shipment of isolates to central reference laboratories might cause undue delays. This might aid in making timely decisions on case management and infection control measures. Patients with suspected PVL-associated disease should be asked for a history of travel or migration because all PVL-MRSA strains observed, as well as the most common MSSA lineage, are known to be locally common in other parts of the world. More than half of PVL-positive isolates were MRSA. In case of hospitalization, patients with suspected PVL-associated disease should, therefore, be treated as MRSA cases until proven otherwise.

## Figures and Tables

**Table 1:** Strain affiliations, PVL- und *mecA* status of study isolates.

Category	CC	Strain	N	Percent
**PVL-negative MSSA**			**35**	**42.2**%
	**CC5**	CC5-MSSA	1	1.2%
	**CC7**	CC7-MSSA	7	8.4%
	**CC10**	CC10-MSSA	2	2.4%
	**CC15**	CC15-MSSA	4	4.8%
		CC15 (ST199)-MSSA	1	1.2%
		CC15 (ST582)-MSSA	2	2.4%
	**CC22**	CC22-MSSA	4	4.8%
		CC22-MSSA-[*ccrAB4*]	2	2.4%
	**CC25**	CC25-MSSA	1	1.2%
	**CC30**	CC30-MSSA	4	4.8%
	**CC45**	CC45-MSSA	4	4.8%
	**CC398**	CC398-MSSA	2	2.4%
	**ST2867**	ST2867-MSSA	1	1.2%
**PVL-negative MRSA**			**9**	**10.8**%
	**CC1**	CC1-MRSA-IV (*aphA3/sat*-positive), “European CC1-MRSA-IV strain”	1	1.2%
		CC1-MRSA-[IV+*fus+ccrAB1*], “WA MRSA-1/45”	1	1.2%
	**CC5**	CC5-MRSA-[IV+*fus+ccrAB*], “Maltese Clone”	3	3.6%
		CC5-MRSA-[VI+*fus*]	1	1.2%
	**CC22**	CC22-MRSA-IV, “UK-EMRSA-15/Barnim EMRSA”	1	1.2%
		CC22-MRSA-[V/VT+*fus*]	1	1.2%
	**CC59**	CC59-MRSA-[V/VT+*fus*]	1	1.2%
**PVL-positive MSSA**			**18**	**21.7**%
	**CC5**	CC5-MSSA (PVL+)	2	2.4%
	**CC6**	CC6-MSSA (PVL+)	1	1.2%
	**CC15**	CC15-MSSA (PVL+)	1	1.2%
	**CC30**	CC30-MSSA (PVL+)	2	2.4%
	**CC88**	CC88-MSSA (PVL+)	1	1.2%
	**CC121**	CC121-MSSA (PVL+)	4	4.8%
	**CC152**	CC152-MSSA (PVL+)	6	7.2%
	**CC772**	CC772-MSSA (PVL+)	1	1.2%
**PVL-positive MRSA**			**21**	**25.3**%
	**CC1**	CC1-MRSA-[V/VT+*fus+ccrAB1*] (PVL+)	1	1.2%
	**CC5**	CC5-MRSA-IV (*sed/j/*r+, PVL+), “Sri Lanka Clone”	4	4.8%
	**CC8**	CC8-MRSA-[IV+Hg] (PVL+), “Spanish/Latin American “USA300”	4	4.8%
		CC8-MRSA-[IV+ACME] (PVL+), „USA300“	1	1.2%
	**CC22**	CC22-MRSA-IV (PVL+/*tst*+)	2	2.4%
	**CC30**	CC30-MRSA-IV (PVL+), “WSPP/Southwest Pacific Clone”	1	1.2%
	**CC88**	CC88-MRSA-IV (PVL+)	1	1.2%
	**CC152**	CC152-MRSA-IV (PVL+)	1	1.2%
		CC152-MRSA-[V/VT+*fus*] (PVL+)	1	1.2%
	**CC398**	CC398-MRSA-V/VT (PVL+)	5	6.0%

**Table 2:** Resistance genes in study isolates. Percentages in brackets refer to the respective group (PVL-negative MSSA and MRSA, PVL-positive MSSA and MRSA).

Gene	Explanation/Corresponding Resistance Phenotype	Total (n = 83)	PVL-Negat. MSSA (n = 35)	PVL-Negat. MRSA (n = 9)	PVL-Posit. MSSA (n = 18)	PVL-Posit. MRSA (n = 23)
***mecA***	MRSA, methicillin/β-lactam	30 (36.1%)	0	9 (100%)	0	21 (100%)
***mecC***	MRSA, methicillin/β-lactam	0	0	0	0	0
***blaZ***	β-lactamase/penicillin	65 (78.3%)	23 (65.7%)	9 (100%)	13 (72.2%)	20 (95.2%)
***fusC***	Fusidic acid (SCC-borne)	9 (10.8%)	0	7 (77.8%)	0	2 (9.5%)
***far1***	Fusidic acid (plasmid-borne)	0	0	0	0	0
***erm*** **(A)**	Macrolides, clindamycin	5 (6%)	0	0	0	5 (23.8%)
***erm*** **(B)**	Macrolides, clindamycin	2 (2.4%)	1 (2.9%)	0	0	1 (4.8%)
***erm*** **(C)**	Macrolides, clindamycin	7 (8.4%)	0	2 (22.2%)	0	5 (23.8%)
***linA/lnu*** **(A)**	Lincosamides, clindamycin	0	0	0	0	0
***msr*** **(A)**	Macrolides	4 (4.8%)	1 (2.9%)	0	1 (5.6%)	2 (9.5%)
***mef*** **(A)**	Macrolides	0	0	0	0	0
***mph*** **(C)**	Macrolides	3 (3.6%)	0	0	1 (5.6%)	2 (9.5%)
***aacA-aphD***	Gentamicin, tobramycin	7 (8.4%)	2 (5.7%)	0	0	5 (23.8%)
***aadD***	Tobramycin	3 (3.6%)	2 (5.7%)	0	0	1 (4.8%)
***aphA3***	Kanamycin	5 (6%)	0	1 (11.1%)	1 (5.6%)	3 (14.3%)
***dfrA***	Trimethoprim	5 (6%)	0	1 (11.1%)	0	4 (19%)
***mupA***	Mupirocin (high level)	2 (2.4%)	1 (2.9%)	0	0	1 (4.8%)
***tet*** **(K)**	Tetracyclines	13 (15.7%)	0	1 (11.1%)	2 (11.1%)	10 (47.6%)
***tet(*** **M)**	Tetracyclines	2 (2.4%)	0	1 (11.1%)	1 (5.6%)	0
***cat* (all variants)** *	Chloramphenicol	4 (4.8%)	1 (2.9%)	0	2 (11.1%)	1 (4.8%)
***cfr***	“PhlopsA” resistance phenotype **	0	0	0	0	0
***fexA***	Chloramphenicol/florfenicol	1 (1.2%)	0	1 (11.1%)	0	0
***vanA***	Vancomycin, teicoplanin	0	0	0	0	0
***qacA/B***	Biozide resistance	0	0	0	0	0
***qacC=smr***	Biozide resistance	3 (3.6%)	1 (2.9%)	0	1 (5.6%)	1 (4.8%)

* For details, see Supplemental file S1; ** “PhlopsA” = Phenicols, Lincosamides, Oxazolidinones, Pleuromutilins, and Streptogramin A.

## Data Availability

All relevant data are provided as Supplementary Materials. A preliminary analysis of the dataset was presented as poster at the annual conference of the German Society for Microbiology and Hygiene in Würzburg, 2024 [92].

## Supplementary Materials

The following supporting information can be downloaded at: https://www.mdpi.com/article/10.3390/pathogens14030238/s1, Supplemental File S1: Lateral flow and microarray test results.

## Abbreviations

The following abbreviations are used in this manuscript:

CC	Clonal complex
MALDI-TOF	Matrix assisted laser desorption ionization (time-of-flight)
MRSA	Methicillin-resistant *Staphylococcus aureus*
MSSA	Methicillin-susceptible *Staphylococcus aureus*
PVL	Panton–Valentine leukocidin
SSTI	Skin and soft tissue infection(s)
ST	Sequence type (as defined by Multilocus Sequence Typing)
